# O-25 PREVENTING TRANSMISSION OF MEASLES IN GOVANHILL, GLASGOW: COMMUNITY ENGAGEMENT WORK TO PROMOTE UPTAKE OF MMR VACCINATION

**DOI:** 10.1093/pubmed/fdag046.022

**Published:** 2026-07-09

**Authors:** MS Helen Benson

**Affiliations:** Public Health Protection Unit, NHS Greater Glasgow And Clyde

## Abstract

**Background:**

In spring 2025 NHS Greater Glasgow and Clyde (GGC) managed a measles outbreak in Govanhill, Glasgow. All 5 notified cases were from the Roma community. None had travelled prior to onset, with no established epidemiological links, suggesting undetected community transmission. Govanhill is an ethnically diverse, multilingual and deprived district including Roma, Eastern European, and South Asian communities with many multigenerational households. Residents may experience barriers in accessing health campaigns and services, and the area is known to have low MMR uptake.

**Objectives:**

To prevent community measles transmission through MMR vaccination.

**Methods:**

Our response included provision of additional opportunities for MMR vaccination. We added a number of additional drop-in MMR clinics and increased capacity of existing clinics. We developed public messaging about measles and how to access MMR vaccination, translated into community languages including video resources sharable across social media. These included recordings in the Romani dialect spoken locally, a spoken-only language. We worked in partnership with peer educators, third sector organisations and community leaders to share these resources.

**Results:**

MMR clinics were held in education, health and community venues, with interpreter support. Though targeted at different age groups, all clinics offered adult and child vaccination. Feedback from community leaders indicated accessible messaging in languages spoken locally was well-received, and clinic attendance was often facilitated by community organisation workers.

**Conclusions:**

Despite high overall rates, several GGC areas have lower MMR vaccination, often associated with deprivation or marginalised groups. With measles increasing in the UK and worldwide, we seek to address this by applying learning from work in Govanhill. With trust in community organisations often higher than in healthcare professionals, key learning was community engagement is as important as ensuring access to healthcare interventions. Key to this is developing appropriate, accessible messaging for local communities and partnership working with local groups.

